# Lipidomic signatures from physically frail and robust older adults at hospital admission

**DOI:** 10.1007/s11357-021-00511-1

**Published:** 2022-02-04

**Authors:** Robinson Ramírez-Vélez, Nicolás Martínez-Velilla, María Correa-Rodríguez, Mikel L. Sáez de Asteasu, Fabricio Zambom-Ferraresi, Sara Palomino-Echeverria, Antonio García-Hermoso, Mikel Izquierdo

**Affiliations:** 1 Navarrabiomed, Hospital Universitario de Navarra (HUN), Navarra Institute for Health Research (IdiSNA), Universidad Pública de Navarra (UPNA), Pamplona, Spain; 2 CIBER of Frailty and Healthy Aging (CIBERFES), Instituto de Salud Carlos III, Madrid, Spain; 3 Department of Nursing, Health Sciences Faculty, University of Granada, Avda. De la Ilustración 60, 18016 Granada, Spain; 4 Laboratorio de Ciencias de La Actividad Física, El Deporte Y La Salud, Universidad de Santiago de Chile, USACH, Santiago, Chile

**Keywords:** Frailty, Lipidomic, Ceramides, Cholesterol, Phosphatidylcholines, Biomarker, Older adults

## Abstract

Identifying serum biomarkers that can predict physical frailty in older adults would have tremendous clinical value for primary care, as this condition is inherently related to poor quality of life and premature mortality. We compared the serum lipid profile of physically frail and robust older adults to identify specific lipid biomarkers that could be used to assess physical frailty in older patients at hospital admission. Forty-three older adults (58.1% male), mean (range) age 86.4 (78–100 years) years, were classified as physically frail (*n* = 18) or robust (*n* = 25) based on scores from the Short Physical Performance Battery (≤ 6 points). Non-targeted metabolomic study by ultra-high performance liquid chromatography coupled to mass spectrometry (UHPLC-MS) analysis with later bioinformatics data analysis. Once the significantly different metabolites were identified, the KEGG database was used on them to establish which were the metabolic pathways mainly involved. Area under receiver-operating curve (AUROC) analysis was used to test the discriminatory ability of lipid biomarkers for frailty based on the Short Physical Performance Battery. We identified a panel of five metabolites including ceramides Cer (40:2), Cer (d18:1/20:0), Cer (d18:1/23:0), cholesterol, and phosphatidylcholine (PC) (14:0/20:4) that were significantly increased in physically frail older adults compared with robust older adults at hospital admission. The most interesting in the physically frail metabolome study found with the KEGG database were the metabolic pathways, vitamin digestion and absorption, AGE-RAGE signaling pathway in diabetic complications, and insulin resistance. In addition, Cer (40:2) (AUROC 0.747), Cer (d18:1/23:0) (AUROC 0.720), and cholesterol (AUROC 0.784) were identified as higher values of physically frail at hospital admission. The non-targeted metabolomic study can open a wide view of the physically frail features changes at the plasma level, which would be linked to the physical frailty phenotype at hospital admission. Also, we propose that metabolome analysis will have a suitable niche in personalized medicine for physically frail older adults.

**Supplementary Information:**

The online version contains supplementary material available at 10.1007/s11357-021-00511-1.

## Introduction

The concept of frailty is gaining traction as an independent clinical syndrome to explain differential outcomes in older adults ^1^. Weakness, slowness, and poor balance are some of the main physical manifestations of frailty, which is associated with adverse events that can lead to a dramatic decline in the quality of life and independence ^2^. It is estimated that 25–50% of all people over the age of 85 are frail and are, consequently, at significantly higher risk of falls, disability, long-term care, and death ^3^.

Frailty is characterized by a host of manifestations, including metabolic abnormalities, neuroendocrine dysregulation, and impaired immunity, and may also be influenced by underlying genetic and environmental factors (e.g., physical inactivity, smoking, and unhealthy diet). Identifying populations at-risk and exploratively identifying potential early and/or predictive biomarkers of age-associated changes that could guide effective health promotion interventions are especially relevant ^4^. One of these strategies has been the investigation of the metabolome, the complete set of molecular weight molecules (metabolites) present in a biological sample. Pujos-Guillot et al. recently used an untargeted metabolomic approach to identify several circulating metabolites that were predictive of pre-frailty ^5^. Metabolomic approaches that analyze changes in metabolite levels thus seem promising for the development of non-invasive diagnostic biomarkers of the frailty phenotype, yet data from older adults at hospital admission are sparse.

The multidimensional nature of frailty presents challenges in accurately measuring the degree of frailty, and several instruments have been developed in recent years to assess the frailty phenotype, although most are focused on different subpopulations of the elderly ^6^. Given the established relationship between functional decline, disability, and comorbidity, an objective clinical approach to assess physical status is to have a “proxy-frailty” measure based on, for example, the Short Physical Performance Battery (SPPB) ^7^. The predictive role of this clinical tool for future disabilities, frailty, and other adverse outcomes including institutionalization, hospital admissions, and mortality has been confirmed in several clinical settings ^7–9^. The SPPB is, however, essentially a descriptive clinical tool and is therefore unable to assess the molecular/biochemical modifications underlying the loss of functionality.

While the causal processes involved in the pathogenesis of frailty are not fully understood, growing evidence supports a role for lipid metabolism in the risk for frailty by modulating skeletal muscle mass and function ^10^. To date, however, few studies have conducted broad lipidomic analyses to assess the relationship between frailty and blood lipids ^11–13^. In the present study, we explored the potential of a targeted lipidomic approach to better characterize the biochemistry underlying physical frailty and to identify molecular biomarkers that might be useful to improve the clinical assessment of frailty in acutely hospitalized older patients at admission. To do this, we compared plasma lipidomic profile differences between physically frail and robust older adults at hospital admission.

## Materials and methods

### Patients and study design

In this study, we enrolled 43 elderly patients from 2018 to 2020 admitted from 2018 to 2020 to an Acute Geriatric Ward of a tertiary Hospital in Spain. Inclusion criteria were age ≥ 70 years, Barthel Index score ≥ 60 points, and ability to ambulate (with/without assistance) and to communicate and collaborate with the research team. Exclusion criteria were very severe cognitive decline (a score of 7 on the Global Deterioration Scale), terminal illness, uncontrolled arrhythmias, acute pulmonary embolism or myocardial infarction, or extremity bone fracture in the past 3 months. Participants signed an informed consent approved by the ethics committee before entering the study. The study followed the principles of the Declaration of Helsinki and was approved by the local Research Ethics Committee (ID Pyto2018/7; 15 May 2018). Patients were evaluated by a staff of 8 geriatricians at the moment of admission and were classified as physically frail (*n* = 18) or robust (*n* = 25) based to their performance on the SPPB.

### Clinical and functional parameters

Height was measured to the nearest 0.1 cm and body mass was measured to the nearest 100 g. Body mass index (BMI) was computed as weight in kilograms divided by height in meters squared. Handgrip strength was measured in the seated position using a Takei 5401 digital dynamometer (Takei Scientific Instruments Co., Tokyo, Japan). Patients were asked to perform 2 maximum force trials for each hand, with the dynamometer beside but not against their body, and the maximum value was used as the final score ^14^.

Physical frailty was measured by the SPPB, which combines balance, gait velocity, and leg strength (five times sit-to-stand test) in a single score from 0 (worst) to 12 (best). Gait speed was calculated for each participant using distance in meters and time in seconds, and was obtained by dividing the distance traveled (4 m) on a flat and unobstructed path by the time to cover that distance. The total SPPB score was obtained through summing the scores obtained from each component. The total score was categorized as follows: 0–6 points = physically frail and 7–12 points = robust. We based this cut-off on two studies. Guimarães Rocco et al. ^15^ highlighted an SPBB cut-off point ≤ 6 as the most suitable for screening physically frail older people in their sample, with a sensitivity of 0.52, a specificity of 0.70, and an accuracy 0.88. Another study, carried out in Spain, also found that the best cut-off point was ≤ 6, with an area under the curve (AUC) of 0.956 and sensitivity and specificity of 0.88 ^9^.

Cognitive function was assessed with the Mini-Mental State Examination (MMSE, 30-point questionnaire; scale of 0 [worst] to 30 [best]) ^16^; and activities of daily living (ADLs) was assessed with the Barthel Index, with a scale of 0 (severe functional dependence) to 100 (functional independence) ^17^. Data related to number of diseases, cumulative illness rating scale score for geriatrics (CIRS-G), and length of hospital stay were collected from clinical records.

### Lipid extraction and UHPLC-MS analysis

Fasting venous blood samples were collected on the next morning after admission to the hospital from the antecubital vein (08:00 to 09:00 am). Blood was inverted five times and allowed to sit for 30 min for clotting. Samples were then centrifuged at 2,000 × g for 10 min at 4 °C, and plasma was aliquoted and stored at − 80 °C until use. Metabolite extraction has been previously described in detail ^22^. Briefly, plasma extracts were mixed with sodium chloride (50 mM) and chloroform/methanol (2:1) in 1.5 mL microtubes on ice. After brief vortex mixing, the samples were incubated for 1 h at − 20 °C. After centrifugation at 16,000 × g for 15 min at 4 °C, the organic phase was collected and dried in a speed vacuum dryer. Dried extracts were reconstituted in acetonitrile/isopropanol (1:1), centrifuged (18,000 × g for 5 min at 4 °C), and transferred to vials for ultra-high performance liquid chromatography coupled to mass spectrometry (UHPLC-MS) analysis. Chromatographic separation and mass spectrometric detection conditions have been previously described ^22^. The UHPLC-MS-based platform was developed for optimal profiling of glycerolipids (di- and tri-glycerides), cholesteryl esters, sphingolipids (ceramides and sphingomyelins), and glycerophospholipids (diacylglycerophospholipids and 1-ether, 2-acylglycerophospholipids).

### Data pre-processing

Data pre-processing was carried out using the TargetLynx application manager for MassLynx 4.1 (Waters Corp., Milford, MA). The chromatography/MS features (as defined by retention time and mass-to-charge ratio pairs, Rt-m/z) used in the study were identified prior to the analysis, either by comparison of their accurate mass spectra and chromatographic Rt with those of available reference standards, or when these were not available, by accurate mass MS/MS fragment ion analysis. Lipid nomenclature and classification follow the LIPID MAPS convention (https://www.lipidmaps.org). Additionally, once the features with significantly different levels (*P*_adjus_ ≤ 0.05) between the study groups were identified, it was possible to establish the most involved metabolic pathways in the study. This approach was performed using the Client-side REST access to KEGG (KEGGREST) package (https://www.genome.jp/kegg/).

### Statistical analysis

Clinical and functional characteristic data for continuous variables are presented as mean (SD). Data normalization was performed by inclusion of multiple internal standards and pool calibration samples following a previously described procedure ^23^. Briefly, logarithmic transformation of the lipidomic data was applied to transform non-normal data into data that follow a normal distribution. *Limma* package (3.34.9) was used to compute the differential expression on physically frail and robust samples ^18^. In addition, data was adjusted for age, sex, and BMI, in order to avoid the influence of confounding variables. Furthermore, lmFit function was used to perform the empirical Bayes (eBayes) statistics and a false discovery rate (FDR). The *P*-values obtained were adjusted according to the Benjamini and Hochberg method ^19^. The adjusted *P*-values < 0.05 were considered significant. Principal component analyses (PCA) between the study group were performed. Later, for each of these compounds, the FoldChangeLog was calculated to select the features with higher differences in plasma levels between the groups. These calculations were performed using the statistical software package R v.3.4.1 (R Development Core Team, 2017; http://cran.r-project.org).

Area under receiver-operating curve (AUROC) analysis was used to test the discriminatory ability of lipid biomarkers for frailty based on the SPPB. Cut-off points were chosen based on the Youden index (*J*), which uses the point on the ROC curve that is farthest from the line of equality ^20^. Validity measurements were calculated for the cut-off point of the SPPB scale and served as a basis for calculating the prevalence of physical frailty and the post-test probability. The positive likelihood ratio was also determined. AUROCs were compared by a non-parametric approach suggested by DeLong et al. ^21^. Percent improvement in prediction error was calculated according to the following formula: 100 × (AUROC_Test-Score-AUROCReference-Score_) / 1 − AUROC_Reference-Score_), where Test-Score refers to the first (superior) and Reference-Score to the second (inferior) score. A *P*-value < 0.05 was generally considered statistically significant. The STATA statistical package (Version 13.0, College Station, TX) was used to perform clinical data management and AUROC analyses.

## Results

The clinical and functional characteristics of the study group are shown in Table 1. The study included 43 older adults (58.1% male) with a mean (SD) age of 86.4 (4.2) years (range, 78–100 years). As expected, the scores for SPPB, gait speed, and five times sit-to-stand test were all significantly lower in the physically frail group than in the robust group, whereas the opposite was seen for functional dependence.

**Table 1:** Clinical and functional characteristics at hospital admission

Variable	Full sample *n* = 43	Physically frail *n* = 18	Robust *n* = 25	*P* for group
Sex (male) (*n* (%))*	25 (58.1)	15 (83.3)	10 (40.0)	0.472
Age (years)	86.4 (4.3)	87.8 (3.9)	85.4 (4.3)	0.075
Body mass (kg)	69.0 (15.9)	71.4 (12.9)	67.2 (17.8)	0.401
BMI (kg/m^2^)	27.5 (5.4)	27.2 (5.2)	27.7 (5.6)	0.790
CIRS-G score^b^	11.9 (6.3)	11.0 (7.0)	12.9 (5.4)	0.405
Length of hospital stay (days)	7.2 (2.0)	7.2 (1.2)	7.0 (1.6)	0.560
SPPB (score)^c^	6.1 (2.9)	3.4 (1.3)	8.1 (2.0)	< 0.001
Gait speed (s (6 m))	10.8 (5.1)	13.7 (6.1)	8.8 (2.8)	< 0.001
Five times sit-to-stand test (s)	17.9 (8.5)	24.9 (11.9)	15.1 (4.4)	0.002
MMSE (score^d^)	23.0 (4.2)	22.8 (3.8)	23.1 (4.6)	0.829
Barthel Index (ADL) (score)^e^	76.3 (17.4)	69.7 (16.4)	81.5 (16.7)	0.030
Handgrip strength (kg)	17.6 (5.4)	17.4 (5.4)	17.7 (5.5)	0.853

Data are presented as the mean (SD), except for the sex group*. *BMI*, body mass index; *CIRS-G*, Cumulative Illness Rating Scale for Geriatrics; *SPPB*, Short Physical Performance Battery. ^a^The most prevalent diseases were coronary, pulmonary, genitourinary, and neurologic diseases. ^b^The CIRS-G scale evaluates individual body systems, ranging from 0 (best) to 56 (worst). ^c^The SPPB scale ranges from 0 (worst) to 12 (best). ^d^*MMSE*, The Mini-Mental State Examination ranges from 0 (worst) to 30 (best). ^e^The Barthel Index ranges from 0 (severe functional dependence) to 100 (functional independence)

**Fig. 1:**
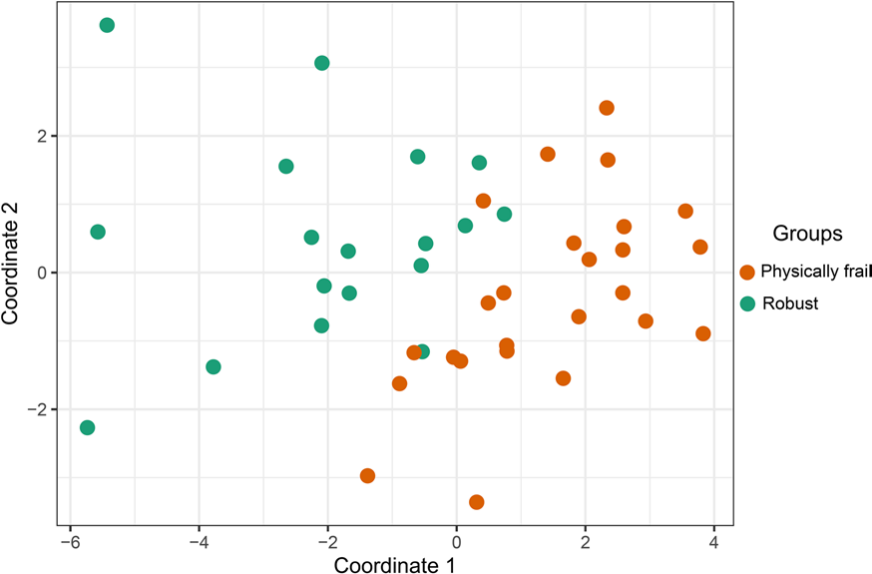
Principal component analysis (PCA) between the study groups. Score plot of PCA using 250 features identified by the metabolomic study between the study groups. The orange points correspond to physically frail subjects, and the green points correspond to and robust subjects

The untargeted metabolomic study of the physically frail and robust plasma samples performed by UHPLC-MS identified 250 common features among the study individuals (Electronic Supplementary Material 1), of which had statistically significant differences between the study groups, after “FDR” adjustment. PCA by the study group dispersion is shown in Fig. 1.

**Fig. 2:**
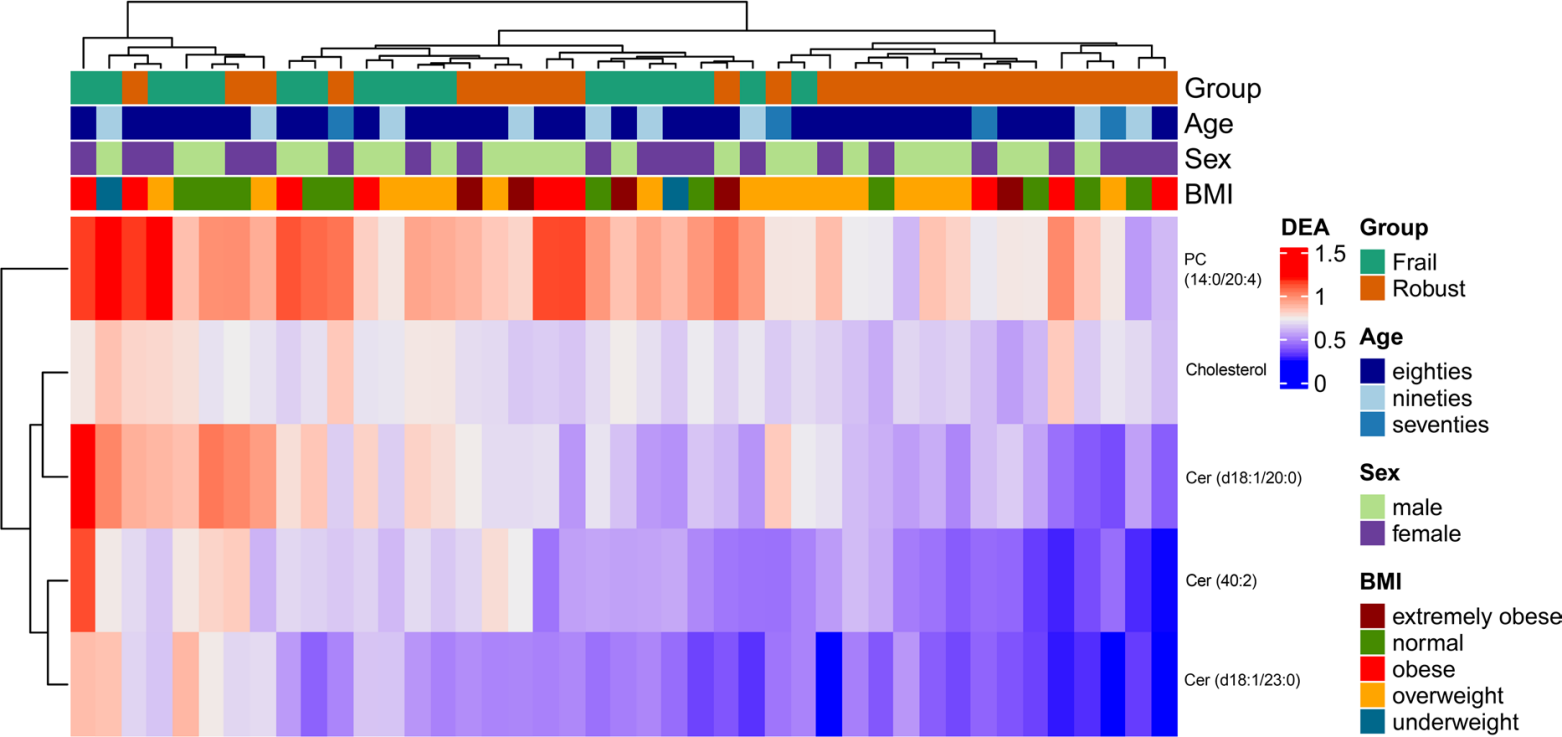
Heatmap of Cer (40:2), Cer (d18:1/20:0), Cer (d18:1/23:0), cholesterol, and PC (14:0/20:4) after logarithmic transformation of the data

**Fig. 3:**
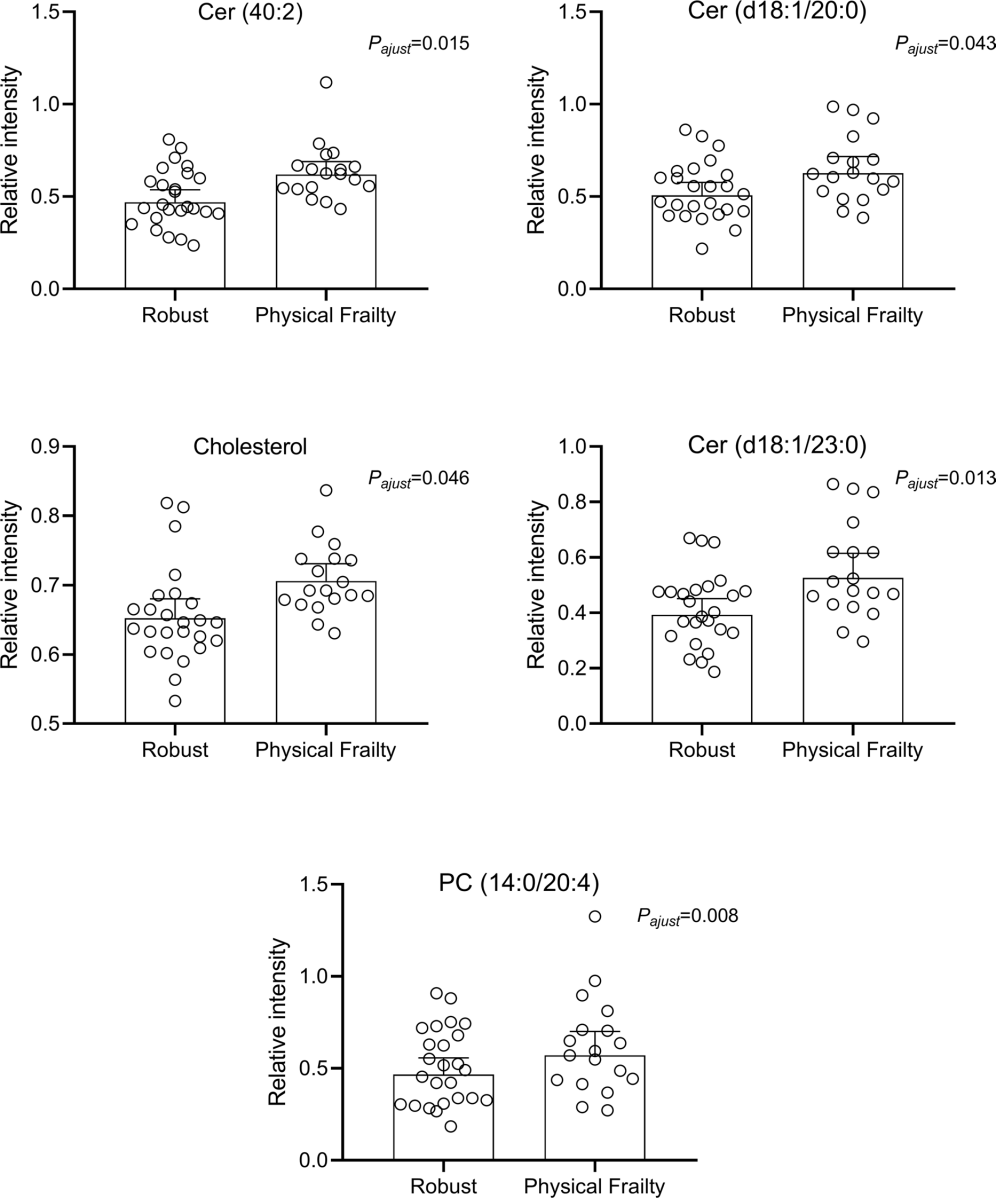
Boxplots of Cer (40:2) (upper left panel), Cer (d18:1/20:0) (upper right panel), Cer (d18:1/23:0) and cholesterol (middle panel), and PC (14:0/20:4) (bottom panel) after logarithmic transformation of the data. FoldChangeLog value for each metabolite was 0.94, 0.87, 0.87, 0.80, and 0.79, respectively

The outcome obtained by this selection criterion is presented in the heatmap displayed in Fig. 2, in which it was possible to isolate five features that were highly differentiated between the study groups. Changes were observed in both neutral lipids and phospholipids, including sphingolipids (ceramides [Cer]), cholesterol, and phosphatidylcholine (PC) which are also displayed in the boxplots of Fig. 3, and were possible to observe in the HMDB and LIPID MAPS database.

Regarding the most involved metabolic pathways in the physically frail metabolome study, it was possible to link features with significant differences between the study groups with 27 different metabolic pathways of the KEGG/HMDB databases. However, only 15% (*n* = 4) of the metabolic pathways were linked by four metabolites; these metabolic pathways are shown in Table 2.

**Table 2:** The metabolic pathways most involved in the physically frail metabolome study

Metabolite (common name)	Chemical formula	KEGG code	KEGG pathways/biological process	KEGG map code
Cholesterol	C_27_H_46_O	C00187	Lipid and atherosclerosis	map05417
			Vitamin digestion and absorption	map04977
			Steroid hormone biosynthesis	map00140/map00100
			Metabolic pathways	map01100
			Fat digestion and absorption	map04975
			Cholesterol metabolism	map04979
			Overview of biosynthetic pathways	map01010
			Bile secretion	map04976
			Primary bile acid biosynthesis	map00120
			Steroid degradation	map00984
			Microbial metabolism in diverse environments	map01120
			Ovarian steroidogenesis	map04913
			Aldosterone synthesis and secretion	map04925
			Cortisol synthesis and secretion	map04927
PC (14:0/20:4)	C_42_H_76_NO_8_P	C00157	Biosynthesis of secondary metabolites	map01110
			Metabolic pathways	map01100
			alpha-Linolenic acid metabolism	map00592
			Linoleic acid metabolism	map00591
			Arachidonic acid metabolism	map00590
			Glycerophospholipid metabolism	map00564
Cer (d18:1/20:0)	C_38_H_75_NO_3_	C00195	Sphingolipid signaling pathway	map04071
			Insulin resistance	map04931
			Sphingolipid metabolism	map00600
			Metabolic pathways	map01100
			Neurotrophin signaling pathway	map04722
			Adipocytokine signaling pathway	map04920
			AGE-RAGE signaling pathway in diabetic complications	map04933
			Diabetic cardiomyopathy	map05415
Cer (d18:1/23:0)	C_41_H_81_NO_3_	HMDB0000950*	Lipid peroxidation	−
			Insulin signaling pathway	−
			Apoptosis	−
			Lipid metabolism pathway	−
			Phospholipid metabolism	−
			Lipid transport	−
			Lipid metabolism	−
			Fatty acid metabolism	−

We used the metabolic pathways by the Kyoto Encyclopedia of Genes and Genomes (KEGG) database were used for searches metabolites by their chemical name in the database and their KEGG codes were registered. This code locates the pathways where the metabolite is involved. Then, the script program obtains the most involved metabolic KEGG pathways by counting the number of metabolites (significantly differentiated) involved in each pathway (https://www.genome.jp/kegg/). *Lipid nomenclature and classification follow the LIPID MAPS convention (https://www.lipidmaps.org) from The Human Metabolome Database (HMDB) ID. –, no informed

**Fig. 4:**
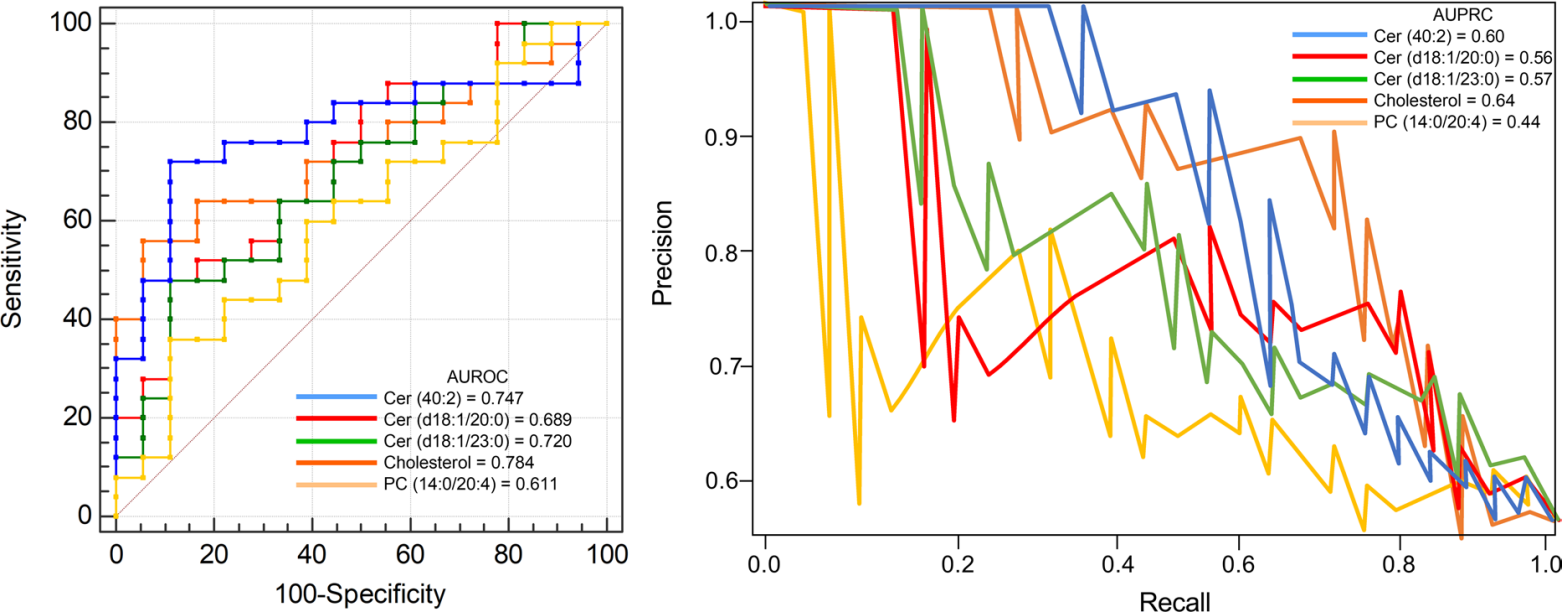
Performance of five plasma metabolites in prediction of physically frail. Receiver operating characteristic (AUROC, left panel) and precision-recall cures (AUPRC, right panel) for different plasma metabolite scores in prediction of physically frail at hospital admission

The AUROC analyses of five plasma metabolites in identification of physically frail at hospital admission are shown in Table 3. The AUROC of the cholesterol with regard to presence of physically frail was similar to Cer (40:2) biomarkers (Fig. 4); however, no differences in AUROCs were also observed.

**Table 3:** Performance of ROC-derived cut-off values for Cer (40:2), Cer (d18:1/20:0), Cer (d18:1/23:0), cholesterol, and PC (14:0/20:4) in identification of physically frail at hospital admission

Parameter	Cer (40:2)	Cer (d18:1/20:0)	Cer (d18:1/23:0)	Cholesterol	PC (14:0/20:4)
AUC (SE)	0.747 (0.0751)	0.689 (0.0825)	0.720 (0.0791)	0.784 (0.0732)	0.611 (0.0885)
95% CI	0.591 to 0.867	0.530 to 0.821	0.562 to 0.846	0.633 to 0.895	0.450 to 0.756
*P*-value	< 0.0001	< 0.0001	< 0.0001	< 0.0001	< 0.0001
Youden index	0.5044	0.3689	0.3689	0.6089	0.2489
Cut-off^a^	0.457	0.4724	0.3862	0.6653	0.3386
Sensitivity	56.00	48.00	48.00	72.00	36.00
Specificity	94.44	88.89	88.89	88.89	88.89
+ LR	10.08	4.32	4.32	6.48	3.24
− LR	0.47	0.59	0.59	0.32	0.72
+ PV	93.3	85.7	85.7	90.0	81.8
− PV	60.7	55.2	55.2	69.6	50.0

*AUC*, area under the curve; *SE*, standard error; *CI*, confidence interval; + *PV*, positive predictive value; − *PV*, negative predictive value; + *LR*, likelihood ratio positive; − *LR*, likelihood ratio negative. ^a^Most suitable threshold according to ROC analysis and Youden’s *J* statistic

## Discussion

Frail older people are highly vulnerable to negative health outcomes such as falls, fractures, disability, and dementia, which is associated with poor quality of life ^22^. Consequently, there is a need for developing new biomarkers to predict frailty in the older population. In the present study, we characterized the plasma lipidomic profiles of physically frail and robust older adults at hospital admission using targeted UHPLC-MS. We found that the circulating plasma levels of five metabolites including ceramides, cholesterol, and phosphatidylcholines were significantly lower in physically frail older adults. Among them, cholesterol and ceramides had acceptable levels of accuracy to discriminate physically frail at hospital admission and, accordingly, might be a useful biomarker in clinical practice.

The development of serum biomarkers for frailty in older adults would be enormously valuable in clinical practice because of its association with premature mortality ^23^ and the inevitable increased cost and use of healthcare resources ^24^. Our analysis revealed a signature of five metabolites including ceramides Cer (40:2), Cer (d18:1/20:0), Cer (d18:1/23:0), cholesterol, and PC (14:0/20:4) that were significantly increased in physically frail compared to robust older adults at hospital admission. Interestingly, a recent metabolome study also showed that some features were linked to some pathophysiological alterations of sarcopenia ^25^.

We identified differential expression of ceramides Cer (40:2), Cer (d18:1/20:0), and Cer (d18:1/23:0), which were all significantly higher in physically frail older adults. Sphingolipids, including ceramides, have their essential biological roles to influence aging ^26^. Ceramides increase in concentration with age in mammals and have been linked to various age‐related ailments, including cancer, type 2 diabetes, neurodegeneration, immune dysfunction, and cardiovascular disease ^26^. Huang et al. also have showed that ceramide accumulation is also correlated with increased insulin resistance and oxidative stress ^27^. Interestingly, Chaurasia et al. found that the deletion of dihydroceramide desaturase 1 improves insulin resistance and hepatic steatosis in mice ^28^. Thus, it has been proposed that clinical therapies that reduce ceramide concentrations may delay or ameliorate symptoms of aging in humans.

Our study also supports that cholesterol, which had a ROC AUC value greater than 0.75 (defined as being of good diagnostic value), might be useful to discriminate physically frail from robust older adults. Several studies have examined the usefulness of different lipids or lipid-related proteins as biomarkers of aging or age-related disease ^26^. Interestingly, this metabolite has been previously associated with hypercholesterolemia and hyperlipoproteinemia ^29,30^, which are well-known cardiovascular risk factors related to atherosclerosis. In addition, due to the role of cholesterol in myelin maintenance and cognition in schizophrenia, it has been considered being potentially relevant in neurological disorders. Al Awam et al. identified cholesterol as a good biomarker to discriminate between schizophrenia patients and unaffected controls ^31^. Among the same line, previous studies have suggested a role for steroid analysis as a potential diagnostic tool ^32–34^. Our findings suggest that cholesterol might be relevant for frailty development. However, since the pathophysiological alterations of cholesterol metabolism associated with frailty are still incompletely understood, further studies are needed to understand the underlying mechanisms for the breakdown in the cholesterol machinery.

We also identified differential expression of PC (14:0/20:4), which was significantly higher in physically frail older adults. Note that this phosphatidylcholine has been previously proposed as an obesity biomarker ^35–37^. Phosphatidylcholine is a major phospholipid of cellular membrane and is a well-known marker of age-related membrane degeneration ^38^. Moreover, it is worth noting that deregulation of lipid metabolism markers such as phosphatidylcholine has been reported to contribute to the link between physical frailty and cognitive decline ^39^.

The results of the present study may have important implications for clinical management and public health surveillance. We identify five biomarkers that could facilitate the stratification of physically frail older adults, providing a clinical resource for diagnosis that supports the importance of lipidomic evaluation in this population. Future longitudinal studies should investigate the potential of these biomarkers as predictors for frailty development. Likewise, given the complexity of frailty phenotype, exploratory approaches will be needed to allow the identification of specific signatures relevant to distinguish frailty status. Of note, metabolic pathways, vitamin digestion and absorption, the AGE-RAGE signaling pathway in diabetic complications, and insulin resistance were linked by four of the metabolites identified in this study. More research is needed to determine the main consequences of the aberrant lipidomic profile in frail older adults and to provide more insight into the metabolic pathways in frailty. The identification of metabolomic profiles of physically frail older adults may allow the design of strategic interventions and approaches for delaying the progression of frailty in older populations, and the demonstration of changes in some lipid metabolomic biomarkers prior to the onset of frailty could provide a basis for future preventative interventions.

Our present study is not without limitations. First, our analysis was cross-sectional, and so causative relationships could not be made. Longitudinal studies will be needed to determine temporal relationship between changes in the lipidomic profile and the development of physical frailty. Second, the study population comprised a cohort including only Caucasians, which impedes the generalization of our findings to other ethnic groups. Additionally, although the cohort was extensively characterized, it was relatively small and analyses involved a large set of variables. Even considering these limitations, the study included 250 metabolic features and UHPLC-MS was used for the analyses, which has been established as a sensitive and highly reliable method for lipidomic analysis ^40^. Also, we investigated, for the first time to our knowledge, the correlation between serum lipid changes in physically frail and robust patients using a targeted lipidomic method, which could be a good starting point to further investigate the predictive value of the specific lipidomic signatures of frailty.

## Conclusion

In summary, the present targeted lipidomic approach found that five circulating metabolites including ceramides, cholesterol, and phosphatidylcholines were significantly increased in physically frail compared with robust older adults at hospital admission, pointing to the existence of a lipidomic profile of older adults with physical frailty. Moreover, cholesterol and ceramides had acceptable levels of accuracy to discriminate physically frail at hospital admission and, therefore, might be useful biomarkers in clinical practice. The non-targeted metabolomic study can open a wide view of the physically frail features changes at the plasma level, which would be linked to the physical frailty phenotype at hospital admission. Also, we propose that metabolome analysis will have a suitable niche in personalized medicine for physically frail older adults. Larger-scale longitudinal studies will be needed as a next step to provide novel insights about aberrant lipid metabolism and the development of physical frailty in older adults.

## Supplementary Information

Below is the link to the electronic supplementary material.

Supplementary Table A1 (xlsx 14.8 KB)

## Data Availability

The data that support the findings of this study are available from the corresponding author upon reasonable request.
